# X marks the spot: catheter aspiration using the Inari FlowTriever device to debulk defibrillator lead vegetations prior to transvenous lead extraction—a case report

**DOI:** 10.1093/ehjcr/ytae332

**Published:** 2024-07-11

**Authors:** James Clark, Abbas Zaidi, Peter O’Callaghan, Ulrich von Oppell, Andrew S P Sharp

**Affiliations:** Department of Cardiology, University Hospital Wales, Cardiff CF14 4XW, UK; Department of Cardiology, University Hospital Wales, Cardiff CF14 4XW, UK; Department of Cardiology, University Hospital Wales, Cardiff CF14 4XW, UK; Department Cardiothoracic Surgery, University Hospital Wales, Cardiff CF14 4XW, UK; Department of Cardiology, University Hospital of Wales and Cardiff University, Cardiff CF14 4XW, UK

**Keywords:** Vegetation, Aspiration, CIED, Defibrillator, Endocarditis, Case report

## Abstract

**Background:**

When cardiac implantable electronic device infection occurs, standard therapy is usually total system extraction. Transvenous lead extraction is preferable to open heart surgical extraction, unless contraindicated because of the presence of very large vegetations on the intravenous leads according to the European Society of Cardiology guidelines. Extraction of transvenous leads with vegetations risks distal embolism resulting in obstruction and/or infection in the pulmonary arteries. Catheter aspiration of vegetations or thrombi has been performed prior to transvenous lead extraction using a partial veno-venous extracorporeal bypass circuit. We report the use of a single-access aspiration system using the Inari FlowTriever 24 French system to debulk a defibrillator lead before percutaneous extraction.

**Case summary:**

A 79-year-old male presented with fever 18 years after his first implantable cardioverter defibrillator implant and 9 years after his most recent pulse generator change. Two large vegetations were identified on his transvenous defibrillator lead on the atrial aspect, near the tricuspid annulus, which were aspirated using the Inari Medical 24Fr FlowTriever aspiration catheter. We describe anatomical considerations during the approach and a technique to localize the vegetations based on a combination of fluoroscopy and transoesophageal echocardiogram guidance.

**Discussion:**

This case demonstrates the safe and effective use of the Inari Medical 24Fr FlowTriever aspiration catheter in debulking a defibrillator lead before transvenous lead extraction. This method uses a single venous puncture and is not dependent on extracorporeal bypass. Apart from reducing complexity, this technique may be advantageous in patients where anticoagulation needs to be minimised.

**Learning points**

- The Inari Medical 24Fr FlowTriever system can feasibly be used to aspirate intracardiac vegetations or thrombi without the need for a bypass circuit or prolonged anticoagulation prior to transvenous lead extraction.
- When passing a guidewire from the right femoral vein into the pulmonary circulation, it can be used to interact with and identify the crossing point between the tricuspid annulus and the defibrillator lead, as well as provide a ‘safety wire’ to allow access to the pulmonary circulation for aspiration should a vegetation embolize during the attempt.

## Introduction

Standard therapy for patients with cardiac implantable electronic device infection is usually complete system extraction. Open surgical extraction may be required for those with large vegetations, though this is sometimes not possible due to prohibitive peri-operative risks.^1–3^ Percutaneous transvenous lead extraction (TLE) is favoured over open surgical extraction unless contraindicated, such as in the case of very large vegetations, and differing techniques may be required to prevent the potentially fatal complication of distal embolization, which occurs more frequently in patients with larger vegetations.^3,4^ There is growing evidence (expert opinion) supporting prior percutaneous debulking of large vegetations.^4^ Even small masses (<2 cm) have been seen to embolize during TLE, where debulking with catheter aspiration has not been utilised pre-operatively.^5,6^ To our knowledge, this is the first use in the UK of the Inari Medical 24Fr FlowTriever system to debulk lead vegetations through a single vascular access point, preventing the need for dual access veno-venous bypass circuit. The Inari Medical 24Fr FlowTriever aspiration system is indicated for the non-surgical removal of thrombi or emboli in vasculature and has been utilized to debulk tricuspid valve endocarditis in patients after veno-venous extra corporeal membrane oxygenation.^7^

## Summary figure

**Figure:**
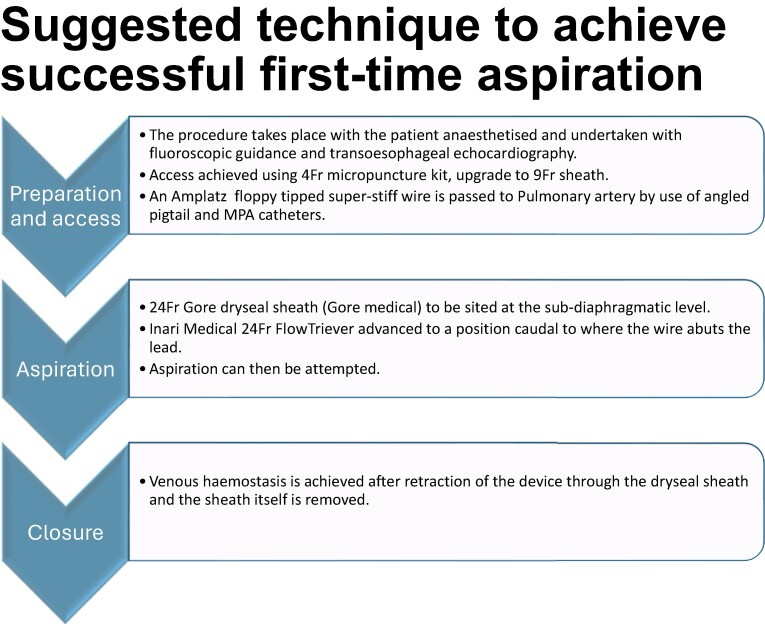


## Case report

We present a case describing catheter aspiration of two vegetations in a 79-year-old male being treated for endocarditis of his transvenous defibrillator lead.

The patient underwent implantation of a secondary prevention implantable cardioverter defibrillator in 2006 when he presented with collapse in consequence to a sustained monomorphic ventricular tachycardia. He had previously suffered an inferior myocardial infarction resulting in moderate left ventricular systolic dysfunction. He underwent a routine generator change in 2015. His routine medications included ramipril 5 mg, bisoprolol 1.25 mg, aspirin 75 mg, and atorvastatin 20 mg.

On the present admission, in 2024, the patient described a 48 h history of fever and complained of pain and swelling at the site of his pre-pectoral pocket. Fever was recorded at 38.9°C, and the implant site was inflamed and tender.

**Figure 1:**
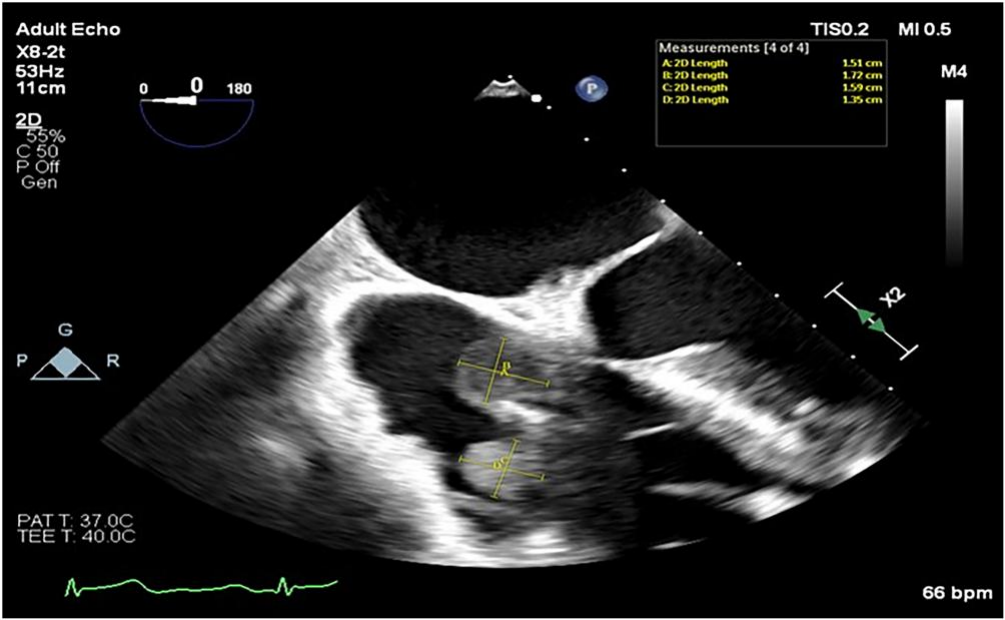
The dimensions of both mobile masses adhered to the defibrillator lead at the level of the tricuspid annulus. (*A*) 15 mm, (*B*) 17 mm, (*C*) 16 mm, and (*D*) 14 mm.

Blood cultures were collected over 7 days, and antimicrobials were initially targeted at likely infective endocarditis (IE) organisms and sequentially altered to cover an ever-broader spectrum after failure to achieve therapeutic response. Transoesophageal echocardiography demonstrated two spherical mobile masses, one 15 mm and the other 17 mm in diameter, adherent to the atrial side of the defibrillator lead just before it crossed the tricuspid valve. *Figure 1* demonstrates the relationship between these structures and the dimensions. He was given a presumptive diagnosis of IE when classified against the modified Duke criteria for IE despite initial negative blood culture results.

In the context of a worsening septic picture and after heart team discussion, the patient was offered an elective attempt at vegetation aspiration, with the aim of debulking the leads prior to percutaneous lead extraction.

The case was conducted under general anaesthesia using fluoroscopic imaging and transoesophageal echocardiography. The lead operator, a cardiac physician, used a 4Fr micro puncture kit (Cook Medical) to access the right common femoral vein under ultrasound guidance. A J-wire was passed to allow a 9Fr sheath to be placed. An Amplatz 7 cm floppy tipped super stiff wire (Boston Scientific) was advanced to the superior vena cava allowing a 24Fr Gore DrySeal Sheath (Gore Medical) to be sited at the sub-diaphragmatic level. This then allowed the passage of the Inari Medical 24Fr FlowTriever to low right atrium (RA).

**Figure 2:**
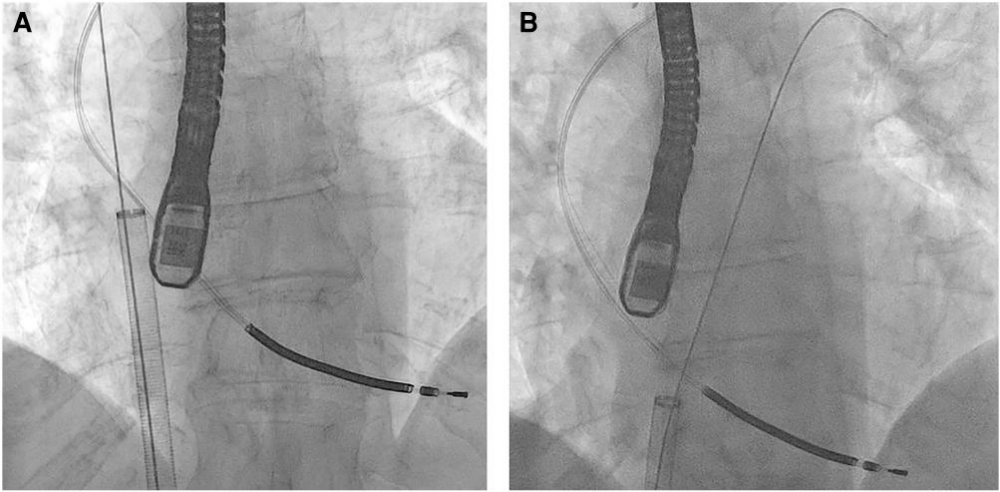
(*A*) Antero-posterior projection of the aspiration catheter in the first position (mid right atrium). (*B*) The aspiration catheter in the same view, at the point of successful aspiration where the wire marks the point where the lead crosses the tricuspid annulus.

The first attempt at aspiration was in the mid RA as seen in *Figure 2A*. This position was chosen as orthogonal transoesophageal echocardiogram and fluoroscopic views implied that the aspiration port was directed towards the vegetation. As per the Inari FlowTriever aspiration method, 60 mL of blood was aspirated. Analysis of the retrieved sample yielded no macroscopic thrombi or vegetations. The operators elected not to risk returning potentially infected microscopic material despite a blood filtering and return system being available for the Inari system.

It was clear that the Inari device was in the wrong plane; therefore, an angled pigtail was passed to the left pulmonary artery and exchanged for a multipurose angiographic catheter, allowing passage of a 1 cm floppy tipped Amplatz super stiff wire (Boston Scientific) to the left pulmonary artery. This wire crossed and abutted the defibrillator lead at the tricuspid annulus where the vegetations had been identified. *Figure 2B* demonstrates the fluoroscopic view of this. The Inari Medical FlowTriever device was advanced over the wire to 1 cm caudal to this crossing point. Aspiration at this point instantly removed both prominent vegetations from the defibrillator lead. Transoesophageal imaging could no longer detect any intracardiac masses.

**Figure 3:**
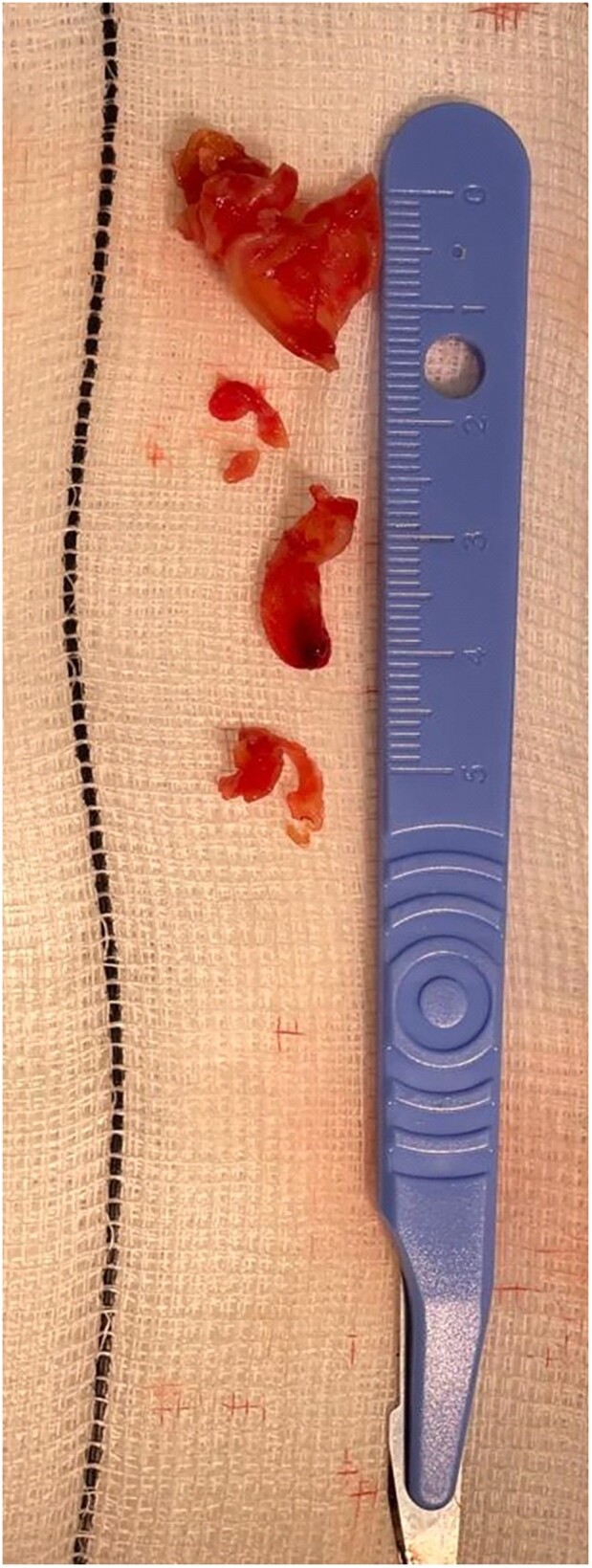
The material aspirated by the Inari Medical FlowTriever system and recovered from the aspiration container.

**Figure 4:**
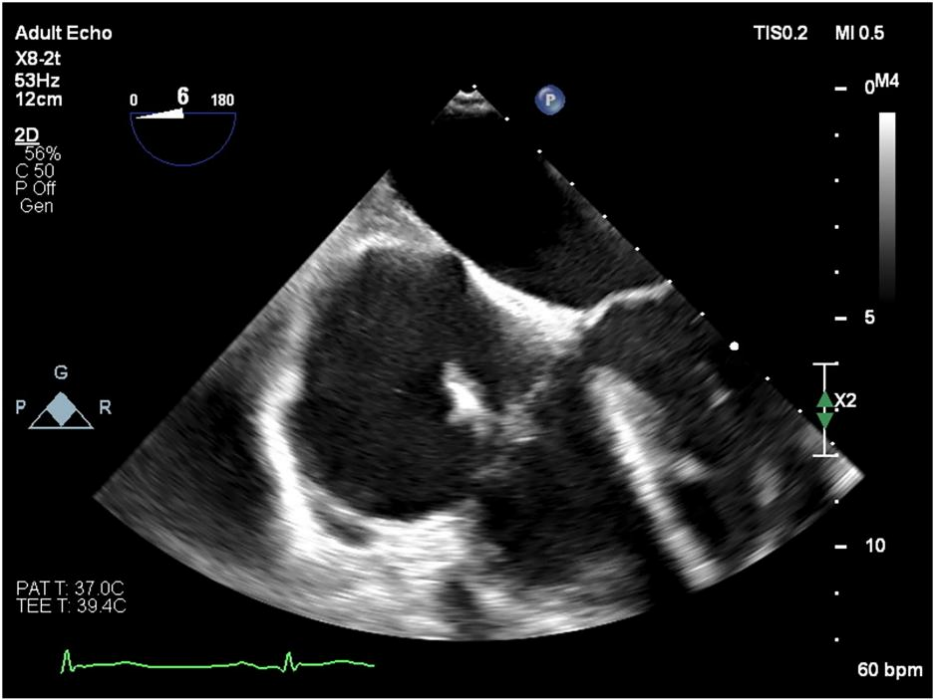
The defibrillator lead has been successfully debulked by removing the two mobile masses.

Multiple masses were then recovered from the aspiration syringe as seen in *Figure 3*. *Figure 4* demonstrates the defibrillator lead with both mobile masses no longer in evidence.

After retraction and removal of the Inari Medical 24Fr FlowTriever, the venous access site was closed with a single Perclose ProGlide (Abbott) suture and manual pressure. The patient went on to have an uncomplicated percutaneous TLE using a rotational cutting device as well as excising the infected device pocket by our centre’s cardiothoracic team. Staphylococci epidermis was later identified by 16 s ribosomal ribonucleic acid sequencing of the vegetation material and the excised pocket material.

The patient has undergone reimplantation of a new device on the contralateral side 3 months after TLE.

## Discussion

This case supports the use of Inari Medical® 24Fr FlowTriever system in the debulking of transvenous defibrillator leads as a preparatory step towards percutaneous TLE in a population who may otherwise require open heart surgery. Previous methods of catheter aspiration have been described, but these have historically required the need for double puncture and partial extracorporeal bypass circuits, a complexity not required by the currently described technique.

In patients with cardiac implantable electronic device endocarditis, concomitant infection of the tricuspid valve and pacing lead occurred in 63% of cases in one epidemiological report.^8^

The siting of a 1 cm floppy Amplatz super stiff wire in the left pulmonary artery was the key to the efficient and safe aspiration of vegetations, ensuring that the aspiration port was directed towards the pacing lead at the point it crossed the tricuspid valve and ensuring rapid access to the pulmonary circulation should accidental distal embolization of material occurs.

Prior use of the AngioVac system (AngioDynamics, Latham, NY) using partial extracorporeal bypass for this purpose has been well described.^9–11^ An analysis of 232 patients found this to be both safe and efficacious.^12^ The Indigo Thrombectomy system (Penumbra, Inc., Alameda, CA) has also been utilized successfully.^13,14^

The use of the Inari Medical® 24Fr FlowTriever system holds the potential advantage in selected patients of a more efficient, single-access procedure, with reduced volumes of systemic anticoagulation required. In our procedure, a single dose of heparin at 70 units per kilogramme body weight was administered. The 24 French aspect of the device allows for aspiration of larger intracardiac masses than the Penumbra Indigo system that is currently available in Europe, though the 16Fr Lightning Flash computer-assisted continuous aspiration system, currently available in the USA, may also prove suitable when available internationally.^12–14^

Current European Society of Cardiology guidelines stipulate that after signs, symptoms, and microbiological evidence of infection are absent for 72 h, or 2 weeks if vegetations or fibrous remnants, reimplantation can proceed at a site distant to the previous generator.^4^

The Inari Medical 24Fr FlowTriever device is a single puncture transvenous aspiration system which can be used to debulk pacing leads before percutaneous TLE without extracorporeal bypass circuits. The use of a floppy tipped stiff wire advanced into the pulmonary vasculature allows easy fluoroscopic identification of where the pacing lead crosses the tricuspid annulus and ensures the aspiration device can be advanced should embolization occurs.

## Data Availability

The data underlying this article are available in the article and in its online supplementary material.

## Lead author biography

James Clark is a cardiology specialist registrar working in South Wales. He is training in heart rhythm.

**Consent:** The authors confirm that informed written consent was obtained for the submission and publication of this case report, including the accompanying text and figures as per COPE guidelines.

**Funding:** None declared.
